# Oscillation Dynamics of Multiple Water Droplets Levitated in an Acoustic Field

**DOI:** 10.3390/mi13091373

**Published:** 2022-08-23

**Authors:** Koji Hasegawa, Manami Murata

**Affiliations:** 1 Department of Mechanical Engineering, Kogakuin University, Tokyo 163-8677, Japan; 2 Graduate School of Engineering, Kogakuin University, Tokyo 163-8677, Japan

**Keywords:** acoustic levitation, droplet, oscillation, levitation stability, droplet–droplet interaction

## Abstract

This study aimed to improve and investigate the oscillation dynamics and levitation stability of acoustically levitated water droplets. Contactless sample manipulation technology in mid-air has attracted significant attention in the fields of biochemistry and pharmaceutical science. Although one promising method is acoustic levitation, most studies have focused on a single sample. Therefore, it is important to determine the stability of multiple samples during acoustic levitation. Here, we aim to understand the effect of multiple-sample levitation on levitation stability in acoustic fields. We visualized the oscillatory motion of multiple levitated droplets using a high-speed video camera. To characterize the dynamics of multiple levitating droplets, the oscillation frequency and restoring force coefficients of the levitated samples, which were obtained from the experimental data, were analyzed to quantify the droplet–droplet interaction. The oscillation model of the spring-mass system was compared with the experimental results, and we found that the number of levitating droplets and their position played an important role in the levitation stability of the droplets. Our insights could help us understand the oscillatory behavior of levitated droplets to achieve more stable levitation.

## 1. Introduction

The acoustic levitation method is a promising technique for manipulating liquid and solid samples in mid-air without any wall effect [1,2,3,4,5]. With this method, contactless acoustic manipulation of samples [6,7,8] has significant implications in material [9,10,11] and pharmaceutical science [12], analytical chemistry [13], and biology [14,15] in terms of preventing heterogeneous nucleation and contamination via container-less processing. However, the strong nonlinearity of the acoustic field causes unsteady oscillatory motion [16,17,18,19,20,21,22,23], interfacial instability [24,25,26,27,28,29,30], resulting in the atomization [31,32,33], and acoustic streaming [34,35,36,37] of a droplet levitated by the acoustic radiation pressure. Therefore, enhancing the levitation stability of a droplet is vital for sample manipulation in acoustic levitation. For the oscillatory motion of a levitated sample, Andrade et al. [18] experimentally revealed the dynamic motion of an acoustically levitated solid sphere and elucidated the particle oscillation dynamics using a simple spring-mass model. Pérez et al. [20] proposed a mass-spring-damper model to understand the oscillatory motion of a solid sphere in the vertical direction and experimentally validated the oscillation model by varying the sound pressure amplitude. In our previous study [22], the effects of sample properties (density, surface tension, and viscosity) on the oscillation dynamics of a single levitated droplet and solid sphere were investigated. We also demonstrated that the oscillation model can predict the natural frequency of droplet oscillation, regardless of the droplet properties. To study their application, Zang et al. [21] investigated the oscillation dynamics of complex droplets containing two immiscible liquids (that is, core-shell-type and non-axisymmetric droplets) in the vertical direction. Hence, they investigated the differences in the damping of oscillations between the two complex droplets.

Although several studies have focused on the oscillation dynamics of a single levitated sample [16,17,18,19,20,21,22,23,24,25,26,27,28,29,30], experimental findings on the oscillation dynamics of multiple levitated droplets in acoustic fields are insufficient. To address this gap, the unsteady oscillatory motion of multiple droplets and the droplet–droplet interaction in an acoustic field were investigated in this study. We focused on the effect of multiple sample levitations on the oscillation of a levitated droplet in an acoustic field. To better understand the droplet dynamics, we quantitatively investigated the characteristics of droplet motion with high-speed visualization and modeled the natural frequency and restoring force coefficient of multiple droplet oscillations. Our findings contribute to the development of contactless droplet manipulation and may inspire further theoretical, numerical, and experimental studies.

## 2. Materials and Methods

### 2.1. Experimental Design

**Figure 1:**
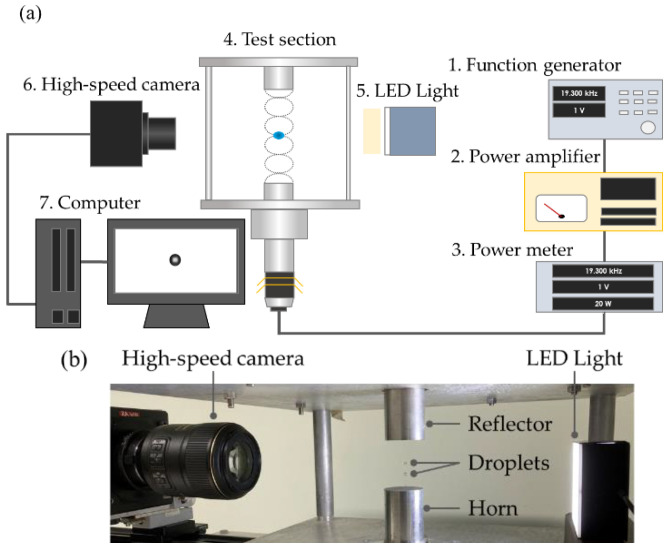

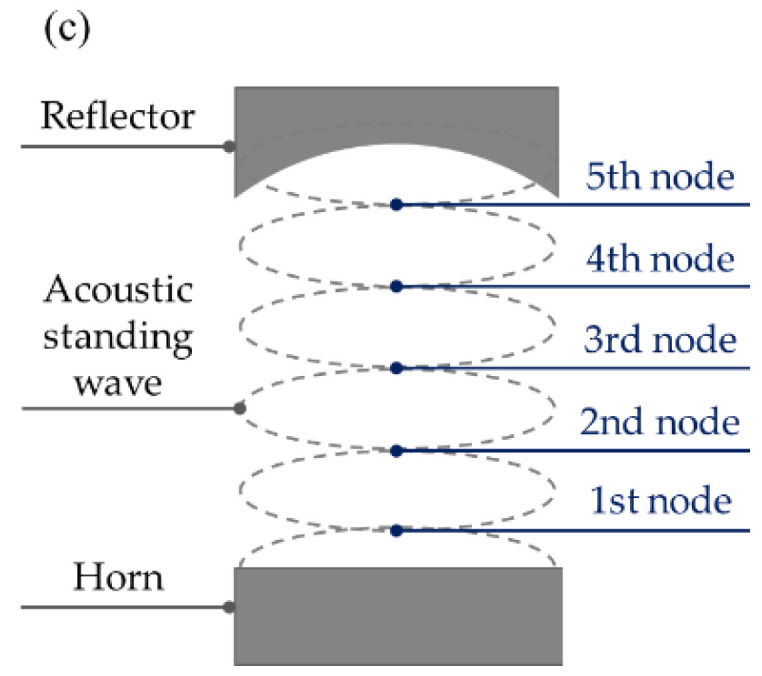
Experimental setup. (**a**) Schematic and (**b**) photograph of acoustic levitation and observation system. (**c**) Acoustic standing wave between the horn (sound emitter) and reflector with five pressure nodes.

Figure 1a,b show a schematic and photograph of the experimental setup, respectively. We used an acoustic levitator system composed of a transducer and reflector that generated a standing wave between the horn–reflector gap. An acoustic wave was generated by the transducer through the bottom horn, which was then reflected at the top reflector surface. The formed acoustic standing wave allowed the droplet to levitate near each pressure node. The 19.3-kHz resonant frequency of this acoustic levitation system was tuned using a function generator (33220A, Agilent Technologies, Inc., Santa Clara, CA, USA) and ultrasonic transducer (D4520PC, NGK SPARK PLUG Co., Ltd., Nagoya, Japan). The ultrasonic transducer was connected to a horn with a diameter of 36 mm. The diameter of the reflector was 36 mm with a 36-mm spherical curvature. The horn–reflector gap was set at approximately 48 mm, which encompassed the five pressure nodes of the acoustic standing wave, as illustrated in Figure 1c. The sound pressure distribution in the horn–reflector gap was measured by a probe microphone (Type 4182, Bryel & Kjaer, Virum, Denmark) with a spatial resolution of 1 mm using a three-dimensional (3D) traversing device. The root-mean-square (RMS) sound pressure was approximately 5 kPa. Pure water was used as the levitated droplet, which had a diameter of 1.5 mm.

The oscillatory behavior of the levitated droplet was captured using a high-speed video camera (FASTCAM-Mini AX200, Photron Co., Ltd., Tokyo, Japan) at 25 °C. The images were recorded at 1000 fps with 1024 × 1024 pixels and a spatial resolution of approximately 30 μm/pixel. From the obtained images, the center of mass of the levitated droplet was analyzed using an in-house MATLAB code to quantify the oscillatory behavior of droplets in an acoustic field. 

**Figure 2:**
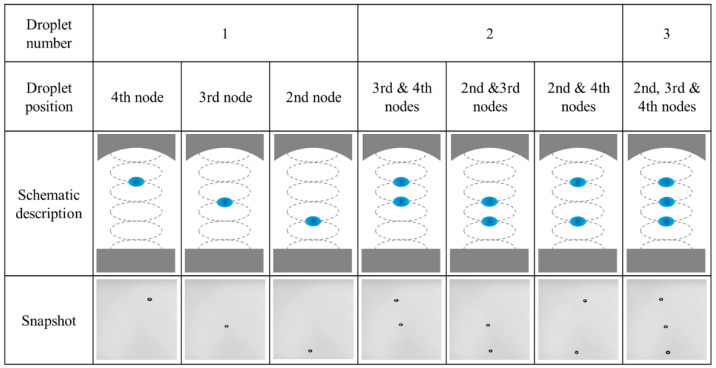
Experimental conditions with single and multiple levitated droplets.

To better understand the effects of droplet–droplet interactions on the oscillation dynamics of levitated droplets, we investigated the effect of the number of droplets and the droplet position in an acoustic standing wave. As summarized in Figure 2, we focused on seven patterns with a combination of various droplet numbers and positions. For a single droplet in an acoustic standing wave, each droplet was levitated at the 2nd, 3rd, and 4th pressure nodes. For two droplets, the droplets were levitated at the 3rd/4th, 2nd/3rd, and 2nd and 4th pressure nodes. For three droplets, the levitated droplets were simultaneously levitated at the 2nd, 3rd, and 4th pressure nodes. 

### 2.2. Theoretical Modeling

A highly effective expression for calculating the acoustic radiation force acting on a small sphere in an acoustic field was described by Gor’kov [4]. The acoustic radiation force *F_rad_* on a small sample (λ >> *R*) can be expressed as follows:

(1) $$F_{rad}=-\nabla U.$$

Here, λ is the wavelength of the sound, and *U* is the Gor’kov potential obtained using

(2) $$U=2\pi R^{3}\left[\frac{\left\langle p_{in}^{2}\right\rangle }{3\rho_{0}c^{2}}-\frac{\rho \langle v_{in}^{2}\rangle }{2}\right],$$

where the angle brackets ⟨ ⟩ represent the time average, *R* is the radius of the sphere, *p_in_* is the acoustic pressure, *ρ*_0_ is the air density, *c* is the speed of sound in air, and *v_in_* is the particle velocity.

In a plane standing wave field, the first-order acoustic pressure and particle velocity distributions between the transducer and reflector can be described by

(3) $$p_{in}=p\sin\left(\omega t\right)\cos\left(kz\right)\;,$$

(4) $$v_{in}=\frac{p}{\rho_{0}c}\sin\left(\omega t\right)\cos\left(kz\right),$$

where *p* is the acoustic pressure amplitude of the sound wave, *ω* (=2π*f*) is the angular frequency of sound, *k* (=2π/*λ*) is the wave number, and *z* is the vertical position. From Equations (3) and (4), the time-averaged acoustic pressure and particle velocity can be expressed as

(5) $$\langle p_{in}^{2}\rangle =\frac{p^{2}}{2}\cos^{2}\left(kz\right),$$

(6) $$\langle v_{in}^{2}\rangle =\frac{1}{2}{\left(\frac{p}{\rho_{0}c}\right)}^{2}\sin^{2}\left(kz\right).$$

By substituting Equations (5) and (6) into Equation (2), the acoustic potential can be rewritten as

(7) $$U=\frac{\pi R^{3}p^{2}}{\rho_{0}c^{2}}\left[\frac{\cos^{2}\left(kz\right)}{3}-\frac{\sin^{2}\left(kz\right)}{2}\right].$$

By substituting Equation (7) into Equation (1), the acoustic force at the pressure node is derived as follows:

(8) $$F_{rad}=\frac{5\pi R^{3}k^{2}p^{2}}{3\rho_{0}c^{2}}z.$$

Moreover, the oscillation of the acoustically levitated sample in an acoustic field can be expressed by the harmonic motion in the spring-mass system. In this case, the acoustic radiation force can be approximated using the restoring force of the spring; therefore, the acoustic radiation force can be described as follows:

(9) $$F_{rad}=k_{z}z,$$

where *k_z_* is the restoring force coefficient (also known as the elastic constant) in the vertical coordinate, and *z_n_* is the position of the *n*th pressure node. Based on Equations (1), (8), and (9), the restoring force coefficients can be calculated as follows:

(10) $$k_{z}=\frac{\partial F}{\partial z}=\frac{\partial^{2}U}{\partial z^{2}}=\frac{5\pi R^{3}k^{2}p^{2}}{3\rho_{0}c^{2}}.$$

Furthermore, the natural frequency of the sphere’s oscillation can be derived using

(11) $$f_{z}=\frac{1}{2\pi }\sqrt{\frac{k_{z}}{m}}=\sqrt{\frac{5k^{2}p^{2}}{4\rho_{0}\rho_{s}c^{2}}},$$

where *m* (=*ρ_s_V*) is the mass of the sample, *ρ_s_* is the density of the sample, and *V* (=4π*R*^3^/3) is the sample volume. Using Equations (10) and (11), we can quantify the restoring force coefficients and oscillation frequency from the experimental conditions of this study, which can be compared with the oscillatory behavior of the levitated droplet captured by the high-speed camera.

## 3. Results and Discussion

### 3.1. Oscillatory Behavior of Levitated Droplets

**Figure 3:**
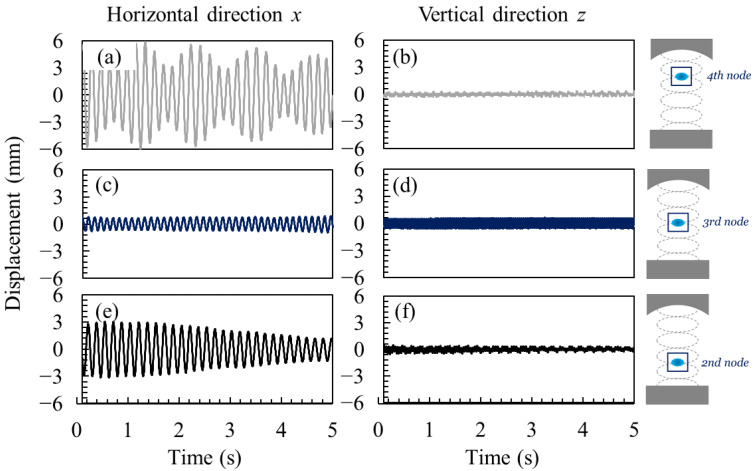
Oscillatory behavior of a single water droplet in an acoustic field. (**a**) Horizontal and (**b**) vertical oscillation of the droplet near the 4th node (top). (**c**) Horizontal and (**d**) vertical oscillation of the droplet at the 3rd node (center). (**e**) Horizontal and (**f**) vertical oscillation of the droplet at the 2nd node (bottom).

Figure 3 shows the time variation of the displacement of a droplet in the horizontal and vertical directions over 5 s, when a single water droplet was levitated in a resonant acoustic field. The droplet was levitated at the 4th (Figure 3a,b), 3rd (Figure 3c,d), and 2nd (Figure 3e,f) pressure nodes. A schematic diagram of each case is shown on the right side of each figure. The results showed that the droplet oscillated periodically. In all cases, the displacement of the droplet in the horizontal direction was larger than that in the vertical direction. This indicates that the sound pressure distribution can be formed vertically in this system such that the droplet is more unstable in the horizontal direction than in the vertical direction. Note that the amplitude of the RMS displacement over time was not constant in the horizontal direction, indicating that the droplet oscillation had multiple frequency components.

**Figure 4:**
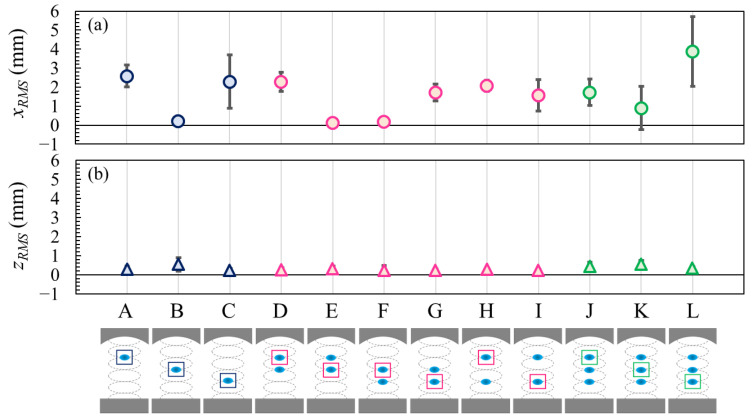
RMS displacement of the levitated droplets in the (**a**) horizontal and (**b**) vertical directions. The average values were used for each plot, and the error bars represent the standard deviations of the three experiments. The solid line represents *x_rms_* = *z_rms_* = 0.

Figure 4 shows the RMS displacements of single, two, and three levitated droplets in the horizontal *x_rms_* and vertical *z_rms_* directions over 5 s. As depicted at the bottom of the figure, we studied 12 cases marked from A to L. Comparing the RMS displacement in the horizontal and vertical directions, the oscillation in the horizontal direction was larger than that in the vertical direction, as shown in Figure 3. 

Regarding the effect of the droplet position, the smallest oscillation in the horizontal direction was confirmed for levitation at the 3rd pressure node, as shown in cases B, E, F, and K. This suggests that the oscillation of the levitated droplet at the 3rd pressure node was suppressed in terms of horizontal motion and was more suitable than levitation at the 2nd or 4th pressure node. The droplets levitated at the 2nd pressure node (cases C, G, I, and L) were more unstable than other droplets. It can be presumed that the sound pressure distribution near the horn is relatively uneven compared to those around the 3rd and 4th pressure nodes [22]. 

As for the effect of the number of droplets, the standard deviation of the horizontal displacement with three droplets (case K) was four times larger than that of a single droplet (case B) or two droplets (cases E and F) in the case of levitation at the 3rd pressure node. We believe that this is due to the droplet–droplet interaction in the sound pressure distribution.

### 3.2. Oscillation Frequency of the Levitated Droplets

**Figure 5:**
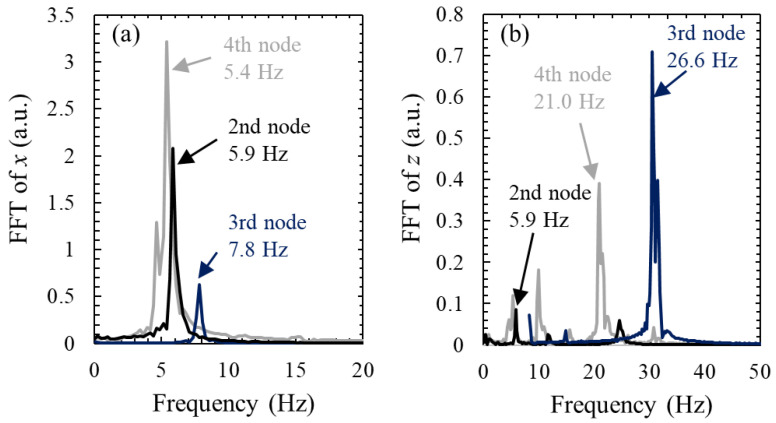
FFT analysis for the (**a**) horizontal and (**b**) vertical oscillations of three levitated droplets. The arrow represents the primary frequency of the corresponding case.

To model the oscillatory behavior of the droplets, a fast Fourier transform (FFT) analysis was applied to identify the natural frequencies of the horizontal and vertical motion of the droplets. The natural frequency plays a critical role in estimating the restoring force coefficient of a droplet to characterize the droplet oscillation. An example analysis of the experimental results for three droplets is shown in Figure 5. The primary frequencies (each maximum value) obtained by FFT are used as natural frequencies in the following discussion. For FFT in the horizontal direction, the primary peak was observed at approximately 5.9, 7.8, and 5.4 Hz for the 2nd, 3rd, and 4th pressure nodes, respectively. For FFT in the vertical direction, the primary peak was observed at 5.9, 26.6, and 21.1 Hz for the 2nd, 3rd, and 4th pressure nodes, respectively. Although the RMS displacement was smaller for the droplet at the 3rd pressure node than the droplets at the 2nd and 4th nodes, the FFT results showed that the natural frequency of the droplet at the 3rd pressure node was the highest. Based on Equations (9) and (11), this indicates that the restoring force of the droplet at the 3rd pressure node can be larger (the droplet is more stable) than that in the other cases. 

**Figure 6:**
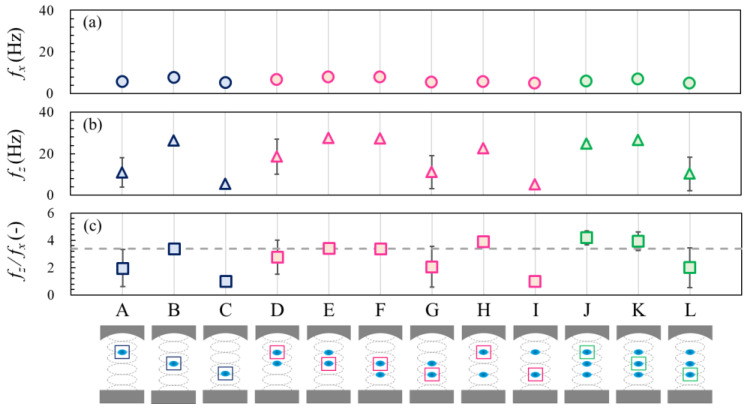
FFT results for all cases. The primary frequency for the (**a**) horizontal and (**b**) vertical oscillations. (**c**) Frequency ratio for each case. The average values were used for each plot, and the error bars represent the standard deviations of the three experiments. The dashed line represents the calculated value in a previous study. Data from [18].

Figure 6 shows the natural frequencies of droplet oscillation for all cases, as in Figure 4. Comparing the natural frequency in the horizontal *f_x_* and vertical *f_z_*, directions in Figure 6a,b, the natural frequency in the vertical direction tended to be larger than that in the horizontal direction. For the droplet at the 3rd pressure node, the natural frequency in the vertical direction was approximately 27 Hz. Using Equation (11) with *k* = 351 1/m, *p_0_* = 5.0 kPa, *ρ_0_* = 1.18 kg/m^3^, *ρ_s_* = 997 kg/m^3^, and *c* = 346.5 m/s for water droplets, the theoretical natural frequency was found to be approximately 26 Hz. These results showed excellent agreement with the theoretical prediction for the droplet at the 3rd pressure node. Furthermore, based on Equations (9)–(11), we can assume that a higher restoring force (stronger spring) was exerted on the levitated droplet in the vertical direction. Regarding the effect of the droplet position, it is clear that the natural frequencies of the droplet at the 3rd pressure node were higher than those at the 2nd and 4th pressure nodes. 

Additionally, we introduced the natural frequency ratio *f_z_*/*f_x_* to characterize the oscillation dynamics of the levitated droplet in Figure 6c. In a previous study [18], the calculated natural frequency ratio was 3.4, as represented by the dashed line in Figure 6c. Our experimental data are in good agreement for the stably levitated droplet at the 3rd pressure node (cases B, E, F, and K). These data demonstrate that the amplitude of the restoring forces in the vertical direction was approximately 3.4 times higher than that in the horizontal direction within the acoustic field, as indicated by the natural frequency ratio; however, this quantitative agreement may be specific to this setup—the generality of these results needs to be verified in the future work. It is worth noting that the displacement of droplet motion in the vertical direction was smaller than that in the horizontal direction due to the higher restoring forces in the vertical direction. This also indicates that the levitated droplets at the 2nd and 4th pressure nodes (case A, C, D, G, I, and L) are relatively less stable compared with the droplet at the 3rd node.

### 3.3. Levitation Stability of Droplets in an Acoustic Field

To better characterize the force field of acoustically levitated droplets, it is critical to quantify the restoring force coefficient, which captures the oscillation dynamics. Based on the results shown in Figure 6, we obtained the restoring force coefficient *k_i_* using Equation (12) derived from Equation (11) with *m* = π*ρ_s_d*^3^/6.

(12) $$k_{i}=m{\left(2\pi f_{i}\right)}^{2}=\frac{2}{3}\pi^{3}\rho_{s}d^{3}f_{i}^{2},$$

where the subscript *i* represents the horizontal *x* or vertical *z* direction, and *d* represents the volume equivalent diameter of the droplet. The natural frequencies in the horizontal and vertical directions are the experimental data shown in Figure 6. 

**Figure 7:**
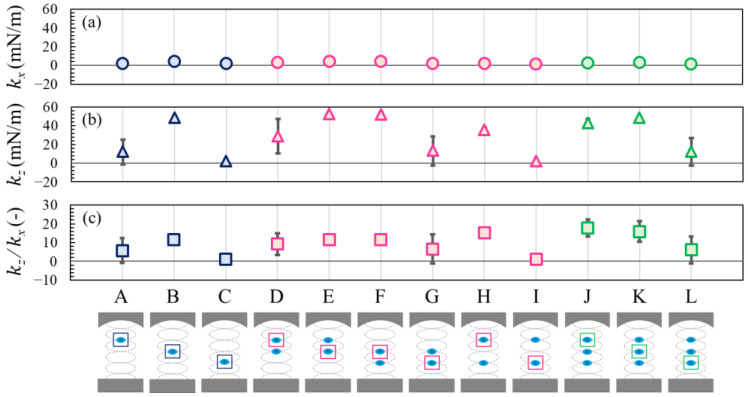
Restoring force coefficient for all cases. (**a**) Horizontal and (**b**) vertical oscillations. (**c**) Restoring force coefficient ratio for each case. The average values were used for each plot, and the error bars represent the standard deviations of the three experiments. The solid line represents *k_x_* = *k_z_* = *k_z_*/*k_x_* = 0.

Figure 7 shows the calculated restoring force coefficients of the levitated droplets in the acoustic field. As indicated by the natural frequencies in Figure 6, the restoring force coefficient in the vertical direction *k_z_* in Figure 7a was larger than that in the horizontal direction *k_x_* in Figure 7b. For the droplet at the 3rd pressure node, the restoring force coefficient in the vertical direction was approximately 50 mN/m using Equation (12); however, when using Equation (10) with *R* = 0.75 mm, *k* = 351 1/m, *p_0_* = 5.0 kPa, *ρ_0_* = 1.18 kg/m^3^, and *c* = 346.5 m/s for water droplets, the theoretical restoring force coefficient was approximately 48 mN/m. These results showed excellent agreement with the theoretical prediction for the droplet at the 3rd pressure node. 

In Figure 7c, we obtained the ratio of the restoring force coefficients *k_z_*/*k_x_*, representing the levitation stability of the sample [17]. Although it is evident from Equation (11), the ratio of the restoring force coefficients is essentially the same as the natural frequency ratio, as shown in Figure 6c, which enables us to estimate the instantaneous force exerted on the levitated sample using Equation (9) and the experimental data of the droplet oscillation. For the restoring force coefficient in the vertical direction *k_z_* of ≈50 mN/m and displacement *z* of ≈0.5 mm, the vertical force on the levitating droplet *F_z_* is roughly 2.5 × 10^−5^ N. For the restoring force coefficient in the horizontal direction *k_x_* of ≈5 mN/m and displacement *x* of ≈2 mm, the horizontal force *F_x_* on the levitating droplet is roughly 1.0 × 10^−5^ N. These estimations show the same order of the force obtained by the numerical simulation [17]. To validate this, it is necessary to measure more accurate and contactless sound pressure and force distributions at the droplet interface using digital holography [31,38]; however, this is beyond the scope of the present study. 

## 4. Conclusions

In summary, we investigated the oscillation dynamics of multiple levitated water droplets during acoustic levitation. We found that the RMS displacement of the oscillatory motion in the vertical direction was significantly smaller than that in the horizontal direction. Furthermore, the ratio of the natural frequencies of the levitated droplets, which was quantified via FFT analysis, was found to be close to 3.4 for the stable levitated droplets at the 3rd pressure node. To better understand the force that acts on the levitating droplets, the restoring force coefficient was derived. The restoring force coefficient can characterize the levitation stability of levitated droplets, and our experimental results agreed well with those of previous analyses. By varying the droplet numbers and positions, we found that the droplet–droplet interaction can affect the levitation stability in a sound field. 

These insights could pave the way for manipulating multiple levitated droplets and enhancing the levitation stability in acoustic levitation; however, the presented results are not completely consistent because the restoring force coefficients must be validated with the measured instantaneous force and sound pressure distribution. Further investigation through contactless measurement of the force and sound pressure distributions around multiple levitated droplets remains a critical challenge. Another key piece of information is the phase difference. If we can characterize the phase differences between droplets oscillated with different frequencies, this would help us understand droplet–droplet interactions. Our findings could provide deeper insight into more stable levitation and manipulation of a levitated sample for potential applications, including 3D displays [39], biological manipulation [40], crystallization [41], and space utilization [42].

## Data Availability

The data presented in this study are available on request from the corresponding author.
